# Risks and benefits of dolutegravir-based antiretroviral drug regimens in sub-Saharan Africa: a modelling study

**DOI:** 10.1016/S2352-3018(18)30317-5

**Published:** 2018-11-29

**Authors:** Andrew N Phillips, Francois Venter, Diane Havlir, Anton Pozniak, Daniel Kuritzkes, Annemarie Wensing, Jens D Lundgren, Andrea De Luca, Deenan Pillay, John Mellors, Valentina Cambiano, Loveleen Bansi-Matharu, Fumiyo Nakagawa, Thokozani Kalua, Andreas Jahn, Tsitsi Apollo, Owen Mugurungi, Polly Clayden, Ravindra K Gupta, Ruanne Barnabas, Paul Revill, Jennifer Cohn, Silvia Bertagnolio, Alexandra Calmy

**Affiliations:** a University College London, London, UK; b Wits Reproductive Health and HIV Institute, Faculty of Health Sciences, University of the Witwatersrand, Johannesburg, South Africa; c Wits Reproductive Health and HIV Institute, University of the Witwatersrand, Johannesburg, South Africa; d University of California, San Francisco, CA, USA; e Chelsea and Westminster Hospital, London, UK; f London School of Hygiene & Tropical Medicine, London, UK; g Brigham and Women's Hospital, Harvard Medical School, Boston, MA, USA; h University Medical Center, Utrecht, Netherlands; i Rigshospitalet, University of Copenhagen, Copenhagen, Denmark; j University of Siena, Siena, Italy; k Africa Health Research Institute, Mtubatuba, South Africa; l University of Pittsburgh, Pittsburgh, PA, USA; m Ministry of Health, Lilongwe, Malawi; n University of Washington, Seattle, WA, USA; o Ministry of Health and Child Care, Harare, Zimbabwe; p HIV i-Base, London, UK; q University of York, York, UK; r Elizabeth Glaser Paediatric AIDS Foundation, Geneva, Switzerland; s World Health Organization, Geneva, Switzerland; t University of Geneva, Geneva, Switzerland

## Abstract

**Background:**

The integrase inhibitor dolutegravir could have a major role in future antiretroviral therapy (ART) regimens in sub-Saharan Africa because of its high potency and barrier to resistance, good tolerability, and low cost, but there is uncertainty over appropriate policies for use relating to the potential for drug resistance spread and a possible increased risk of neural tube defects in infants if used in women at the time of conception. We used an existing individual-based model of HIV transmission, progression, and the effect of ART with the aim of informing policy makers on approaches to the use of dolutegravir that are likely to lead to the highest population health gains.

**Methods:**

We used an existing individual-based model of HIV transmission and progression in adults, which takes into account the effects of drug resistance and differential drug potency in determining viral suppression and clinical outcomes to compare predicted outcomes of alternative ART regimen policies. We calculated disability adjusted life-years (DALYs) for each policy, assuming that a woman having a child with a neural tube defect incurs an extra DALY per year for the remainder of the time horizon and accounting for mother-to-child transmission. We used a 20 year time horizon, a 3% discount rate, and a cost-effectiveness threshold of US$500 per DALY averted.

**Findings:**

The greatest number of DALYs is predicted to be averted with use of a policy in which tenofovir, lamivudine, and dolutegravir is used in all people on ART, including switching to tenofovir, lamivudine, and dolutegravir in those currently on ART, regardless of current viral load suppression and intention to have (more) children. This result was consistent in several sensitivity analyses. We predict that this policy would be cost-saving.

**Interpretation:**

Using a standard DALY framework to compare health outcomes from a public health perspective, the benefits of transition to tenofovir, lamivudine, and dolutegravir for all substantially outweighed the risks.

**Funding:**

Bill & Melinda Gates Foundation.

## Introduction

Scaling up antiretroviral therapy (ART) in sub-Saharan Africa represents a major achievement, but there are ongoing challenges, including increasing transmitted drug resistance,^1^ poor coverage of viral load monitoring,^2^ and low numbers of people who have fulfilled the criteria for failure of first-line ART switching to second-line regimens.^3^

Until recently, WHO recommended a sequence of first-line tenofovir, lamivudine (or emtricitabine), and efavirenz and second-line zidovudine, lamivudine, and a protease inhibitor in people with first-line failure.^4^ However, WHO now recommends use of the integrase inhibitor dolutegravir with tenofovir and lamivudine in people initiating ART and, potentially, in those currently on first-line ART if they have a recent viral load measurement less than 1000 copies per mL.^5^ In people with virological failure on tenofovir, lamivudine, and efavirenz, dolutegravir in the context of an optimised nucleoside reverse transcriptase inhibitor background is recommended as a second-line regimen, which most often will be zidovudine, lamivudine, and dolutegravir. Dolutegravir-based regimens are associated with less need to switch to other antiretroviral drugs and there is a lower risk of development of major drug resistance mutations compared with efavirenz-based regimens.^6^, ^7^ Dolutegravir therapy also has superior outcomes compared with a boosted protease inhibitor-based regimen in people starting second-line therapy with at least one active nucleoside reverse transcriptase inhibitor.^8^ An alternative to this approach to dolutegravir use would be to transition all people on ART to tenofovir, lamivudine, and dolutegravir, unless or until there is sustained virological failure, at which point zidovudine, lamivudine, and a protease inhibitor would be used. Since access to viral load testing is difficult in many countries, this approach has the potential to bring greater public health benefits. However, these benefits should be balanced against concerns over a possible risk of increased resistance to dolutegravir and other drugs. Modelling can help policy makers think through the balance of these considerations in the context of a public health approach.

**Research in context**

**Evidence before this study**

The integrase inhibitor dolutegravir is expected to have a role in future antiretroviral therapy (ART) regimens in sub-Saharan Africa because of its high potency and barriers to resistance, good tolerability, and low cost. However, which policy for use of dolutegravir will produce the greatest population health benefits is unclear because of considerations over the potential for virological failure and drug resistance spread if used with nucleoside reverse transcriptase inhibitors to which HIV is resistant, and a possible association between dolutegravir and an increased risk of neural tube defects in infants if used at conception in women. We searched Web of Knowledge using the terms “dolutegravir” and “model*” on Aug 14, 2018, with no date or language restrictions, and identified 74 papers. When we reviewed these papers we did not identify any that used modelling to quantify the risks and benefits of different policies for introduction of dolutegravir in sub-Saharan Africa.

**Added value of this study**

We used an existing individual-based model of HIV transmission and progression in adults to compare predicted outcomes of alternative regimen policies. The model takes into account the effects of drug resistance and potency in determining viral load and clinical treatment outcomes, and the effect of a potentially increased risk of neural tube defects in infants if dolutegravir is taken by women at conception. We considered policies of using a regimen containing tenofovir, lamivudine, and dolutegravir for all individuals on ART, or of making tenofovir, lamivudine, and dolutegravir dependent on viral load suppression (whether on first-line or second-line ART) or, for women, intention to have (more) children.

**Implications of all the available evidence**

Using a standard disability adjusted life-year framework for comparing health outcomes from a public health perspective, we found that the benefits of transition to a regimen of tenofovir, lamivudine, and dolutegravir for all people on ART, without dependence on viral suppression or intention to have (more) children, substantially outweighed the risks. Our evaluation provides quantitative assessment to guide policy formulation by ministries of health on the use of dolutegravir.

Furthermore, dolutegravir could be a cause of neural tube defects in the children of a small proportion of women taking dolutegravir at the time of conception—in a 2018 report,^9^ four incidences of neural tube defects occurred in 596 pregnancies, which is above the background expected for efavirenz-based therapy regimens. This finding has raised questions over the use of dolutegravir in women of child bearing age. However, restriction of dolutegravir use in women of child bearing age has a public health cost that policy makers should weigh against the possible neural tube defect risk.

In this study, we used an existing individual-based model of HIV transmission, progression, and the effect of ART to help inform policy makers on approaches to the use of dolutegravir that are likely to lead to the highest population health gains. We aimed to provide quantitative assessment of the risks and benefits of alternative policies for use of dolutegravir to inform ministries of health in their policy decision making in consultation with the communities they serve.

## Methods

### Model description

In this modelling study, we used the HIV Synthesis Model (for full details see appendix).^10^ Briefly, this model (programmed in SAS version 9.4) generates a population who are individually tracked, with updates every 3 months, for risk of HIV acquisition. Those who acquire HIV are tracked in terms of their viral load, CD4 cell count, occurrence of WHO stage 3 and 4 conditions, use of specific drugs, presence of resistance mutations, adherence, and drug toxicities. The ongoing effects of a drug regimen (on viral load, drug resistance, and CD4 count, and hence risk of AIDS and death) are dependent on the sum of the activity of each drug in the regimen, accounting for presence of drug resistance mutations, drug potency, and level of adherence. This study was not submitted for ethics committee review as it does not involve research on human subjects.

### Setting scenarios

The model is based on sub-Saharan Africa, with 1000 potential setting scenarios generated through simulation. We randomly varied parameters, including the rate of HIV testing, distribution of ART adherence across individuals, the rate of ART interruption, number of switches to a second-line regimen after failure of a first-line regimen (detected by confirmed viral load above 1000 copies per mL), and the ability to measure viral load as indicated,^11^ within plausible bounds for settings in the region. For a complete list of parameters see the appendix (p 27).

For each setting scenario, we considered the situation in 2018 and compared outcomes of potential regimen policies over a 20 year time horizon (50 years in sensitivity analysis). The regimen policies considered are described in table 1. Our reference regimen policy was a continuation of the approach of use of tenofovir, lamivudine, and efavirenz in first-line regimens. We also considered four further policies, involving use of tenofovir, lamivudine, and dolutegravir. These policies consider tenofovir, lamivudine, and dolutegravir use with or without dependence on intention to have (more) children or current viral load suppression being documented. The rationale for restriction of use of tenofovir, lamivudine, and dolutegravir to those with viral suppression is due to concern that resistance to tenofovir and lamivudine is likely to have developed in those with virological failure, making dolutegravir the only fully active drug and meaning the risk of resistance to dolutegravir is increased. The extent of any residual effects on viral replication due to continued exposure to lamivudine and tenofovir in these circumstances is uncertain.^12^, ^13^ Policies involving tenofovir, lamivudine, and dolutegravir depending on viral suppression also involve use of zidovudine rather than tenofovir in newly initiated second-line regimens. This approach is broadly consistent with current WHO interim guidance.^5^ We refer throughout this Article to use of lamivudine in regimens, but emtricitabine might be used instead. Dependence on a woman's intention to have (more) children relates to concern over the risk of neural tube defects if dolutegravir is used at the time of conception. Policies with this dependence are envisaged to offer dolutegravir to women only upon reaching the point of intention to have no more children. We assume a rate of reaching a point of intention to have no more children to be 0·005 per 3 months from age 25 years. This rate results in 16% of women aged 15–55 years not intending to have (more) children, which is consistent with data from Demographic and Health Surveys.^14^, ^15^ We assume that women who intend to have no more children are able to access and use contraception, and that contraceptive efficacy is 80% (50% in sensitivity analysis). We refer to the policy for which tenofovir, lamivudine, and dolutegravir use is dependent on neither viral suppression nor intention to have more children as tenofovir, lamivudine, and dolutegravir for all.

**Table 1:** Description of regimen policies considered

	**Men and women not intending to have (more) children**					**Women intending to have (more) children**				
	New initiators	Currently on first-line tenofovir, lamivudine, and efavirenz	Currently on second-line zidovudine, lamivudine, and protease inhibitor (atazanavir)	At future tenofovir, lamivudine, and efavirenz failure	At future tenofovir, lamivudine, and dolutegravir failure	New initiators	Currently on first-line tenofovir, lamivudine, and efavirenz	Currently on second-line zidovudine, lamivudine, and protease inhibitor (atazanavir)	At future tenofovir, lamivudine, and efavirenz failure	At future tenofovir, lamivudine, and dolutegravir failure
Tenofovir, lamivudine, and efavirenz for all	Tenofovir, lamivudine, and efavirenz	Tenofovir, lamivudine, and efavirenz	Zidovudine, lamivudine, and protease inhibitor (atazanavir)	Zidovudine, lamivudine, and protease inhibitor (atazanavir)	..	Tenofovir, lamivudine, and efavirenz	Tenofovir, lamivudine, and efavirenz	Zidovudine, lamivudine, and protease inhibitor (atazanavir)	Zidovudine, lamivudine, and protease inhibitor (atazanavir)	..
Tenofovir, lamivudine, and dolutegravir dependent on viral suppression and intention to have (more) children	Tenofovir, lamivudine, and dolutegravir	Switch to tenofovir, lamivudine, and dolutegravir if viral load <1000 copies per mL	Switch to tenofovir, lamivudine, and dolutegravir if viral load <1000 copies per mL	Zidovudine, lamivudine, and dolutegravir	Zidovudine, lamivudine, and protease inhibitor (atazanavir)	Tenofovir, lamivudine, and efavirenz	Tenofovir, lamivudine, and efavirenz	Zidovudine, lamivudine, and protease inhibitor (atazanavir)	Zidovudine, lamivudine, and protease inhibitor (atazanavir)	..
Tenofovir, lamivudine, and dolutegravir dependent on intention to have (more) children only	Tenofovir, lamivudine, and dolutegravir	Switch to tenofovir, lamivudine, and dolutegravir	Switch to tenofovir, lamivudine, and dolutegravir	..	Zidovudine, lamivudine, and protease inhibitor (atazanavir)	Tenofovir, lamivudine, and efavirenz	Tenofovir, lamivudine, and efavirenz	Zidovudine, lamivudine, and protease inhibitor (atazanavir)	Zidovudine, lamivudine, and protease inhibitor (atazanavir)	..
Tenofovir, lamivudine, and dolutegravir dependent on viral suppression only	Tenofovir, lamivudine, and dolutegravir	Switch to tenofovir, lamivudine, and dolutegravir if viral load <1000 copies per mL	Switch to tenofovir, lamivudine, and dolutegravir if viral load <1000 copies per mL	Zidovudine, lamivudine, and dolutegravir	Zidovudine, lamivudine, and protease inhibitor (atazanavir)	Tenofovir, lamivudine, and dolutegravir	Switch to tenofovir, lamivudine, and dolutegravir if viral load <1000 copies per mL	Switch to tenofovir, lamivudine, and dolutegravir if viral load <1000 copies per mL	Zidovudine, lamivudine, and dolutegravir	Zidovudine, lamivudine, and protease inhibitor (atazanavir)
Tenofovir, lamivudine, and dolutegravir for all	Tenofovir, lamivudine, and dolutegravir	Switch to tenofovir, lamivudine, and dolutegravir	Switch to tenofovir, lamivudine, and dolutegravir	..	Zidovudine, lamivudine, and protease inhibitor (atazanavir)	Tenofovir, lamivudine, and dolutegravir	Switch to tenofovir, lamivudine, and dolutegravir	Switch to tenofovir, lamivudine, and dolutegravir	..	Zidovudine, lamivudine, and protease inhibitor (atazanavir)

Assumptions for resistance acquisition with dolutegravir-containing regimens are informed by data on the risk of resistance mutations existing at virological failure and studies of monotherapy, which were mainly done in people with existing viral suppression but include a small study of dolutegravir monotherapy in ART-naive people with viral loads less than 100 000 copies per mL.^6^, ^7^, ^16^, ^17^, ^18^, ^19^, ^20^, ^21^, ^22^ We inferred a 13 times lower rate of resistance to dolutegravir than to efavirenz. Dolutegravir is generally associated with a lower risk of toxicity than are efavirenz and protease inhibitors, resulting in reduced discontinuation.^6^, ^16^, ^19^, ^23^ We assumed that the risk of neurological toxicity with dolutegravir was half that for efavirenz, and assumed that dolutegravir has 1·5 times higher potency than does efavirenz (lower than boosted protease inhibitors, which were assumed to have potency of 2), consistent with its effect as a monotherapy^17^ (although insufficient for its clinical use as monotherapy). We assumed that the residual effects of tenofovir in the presence of the Lys65Arg mutation and lamivudine in the presence of the Met184Val mutation mean that they each have 0·25 times full drug activity in this situation.^12^, ^13^ We present our main results as the mean and mean difference compared with the policy of tenofovir, lamivudine, and efavirenz for all, with a 90% range reflecting variation across setting scenarios and 95% CI reflecting stochastic simulation variability.

Although these assumptions formed our base assumptions, we considered the possibility of different assumptions for dolutegravir in a proportion of our 1000 setting scenarios. These proportions for alternative assumptions were selected on the basis of the perceived probability that they hold. Thus, we considered that potency of dolutegravir could be 1 (20% of setting scenarios), 1·25 (20% of setting scenarios), or 2 times (5% of setting scenarios) that of efavirenz, instead of 1·5 times (55% of setting scenarios); that development of resistance to dolutegravir could be only 3 times lower than for efavirenz (20% of setting scenarios); that the risk of neurological toxicity using dolutegravir could be equal to that of efavirenz (20% of setting scenarios); and that the residual effects of tenofovir and lamivudine in the presence of the Lys65Arg and Met184Val mutations was 0·0 rather than the 0·25 of a fully active drug (20% of setting scenarios in each case). We also considered different degrees to which viral load monitoring was implemented (25% of setting scenarios each for probabilities of 0·0, 0·10, 0·25, and 0·85 of a viral load test being done, and the result delivered in accordance with the recommended monitoring strategy) and ART regimen was switched after viral failure was determined (probabilities of 0·05 in 30% of setting scenarios, 0·20 in 50% of setting scenarios, and 0·50 in 20% of setting scenarios per 3 months). We determined these alternative assumptions by random independent sampling for each setting scenario. Full details of the distribution of all sampled parameters used in deriving our 1000 setting scenarios are shown in the appendix (p 27). Excess risk of neural tube defects in babies of women on dolutegravir at the time of conception was assumed to be 0·58% (4 in 596=0·67% minus the 0·09% background rate in HIV-negative women); we did sensitivity analyses using values of 1% and 3%.

The range of HIV epidemic and programmatic characteristics in 2018 for our 1000 setting scenarios are shown in table 2 with comparable observed data.

**Table 2:** HIV epidemic and programmatic characteristics of setting scenarios in 2018

	Model (2018)*	**Examples of observed data**
HIV prevalence (age 15–49 years)	10% (5–19)	Zimbabwe 2016 14%, Tanzania 2017 5%, Uganda 2017 6%, Lesotho 2017 24%, Swaziland 2017 27%, Malawi 2016 10%†
HIV incidence age 15–49 years (per 100 person-years)	0·8 (0·3–1·6)	Malawi 2016 0·37, Zambia 2016 0·66, Zimbabwe 2016 0·45, Lesotho 2017 1·55, Namibia 2016 0·40, Swaziland 2017 1·48, Tanzania 2017 0·27†
Proportion of HIV-positive people diagnosed	83% (69–93)	Malawi 2016 73%, Zambia 2016 67%, Zimbabwe 2016 74%, Namibia 2017 86%, Tanzania 2017 52%† (see also Kim and colleagues,^24^ which suggests undisclosed diagnosed HIV)
Proportion of all HIV-positive people with viral load <1000 copies per mL	57% (31–71)	Zambia 2016 60%, Malawi 2016 68%, Zimbabwe 2016 60%, Swaziland 2017 73%, Lesotho 2017 68%, Tanzania 2017 52%, Uganda 2017 60%, Cameroon 2017 45%, Namibia 2017 77%†
Proportion of ART-experienced people who have started second-line (boosted protease inhibitors) therapy	2·4% (0·5–9·4)	Malawi 1·5%^25^
Of people on ART, proportion with viral load <1000 copies per mL	85% (74–91)	Zambia 2016 89%, Malawi 2016 91%, Zimbabwe 2016 87%, Cameroon 2017 80%, Namibia 2017 91%, Tanzania 2017 88%, Uganda 2017 84%†
Percentage of ART-naive ART initiators with non-nucleoside reverse-transcriptase inhibitor resistance	11% (2–29)	Angola 2012 14%, Botswana 2016 8%, South Africa 2017 14%, Zimbabwe 2015 10%, Namibia 2015 9%, Cameroon 2015 8%, Uganda 2016 16%^26^
Mother-to-child transmission proportion	3·7% (1·9–6·7)	All 2014: Botswana 2% within 6 weeks, 4% final; South Africa 1% within 6 weeks, 4% final; Namibia 1% within 6 weeks, 7% final; Uganda 2% within 6 weeks, 8% final; Zimbabwe 5% within 6 weeks, 12% final; Malawi 7% within 6 weeks, 7% final; Angola 10% within 6 weeks, 25% final^27^

Values represent 1000 setting scenarios for people aged 15–64 years, unless otherwise stated. ART=antiretroviral therapy.

* Values are median (90% range).

† Data from Population Health Impact Survey.

### Model outcomes

Our primary measure of health outcome was disability adjusted life-years (DALYs). Although we explicitly modelled the individual life courses of adults only, we considered DALY effects of neural tube defects and of mother-to-child HIV transmission. We estimated the aggregate effects that alternative policies have on population burden of disease by calculating net DALYs, which are the DALYs averted by a policy minus the health opportunity costs imposed as a result of costs incurred. Health opportunity costs are calculated using country cost-effectiveness thresholds, which represent the health gains that could be generated by alternative uses of resources.^28^ Country-specific thresholds are uncertain but US$500 averted per DALY is likely to be at the upper end on the basis of evidence concerning how resources would otherwise be used,^29^ and we used this value. We calculated net DALYs as DALYs plus costs divided by the cost-effectiveness threshold. Absolute numbers of health-related events, costs, DALYs, and net DALYs that we report are relevant for a country with a population size of around 10 million adults in 2018. We did our analysis from a health-care perspective. We discounted future costs and health outcomes to present values of 3% per annum. We assumed the costs of tenofovir, lamivudine, and dolutegravir and tenofovir, lamivudine, and efavirenz to be US$75 per year, and the cost of zidovudine, lamivudine, and a protease inhibitor (atazanavir) to be $265.^30^ Full details of unit costs and disability weights are provided in the appendix (p 33).

For a woman having a baby with a neural tube defect, an extra DALY was incurred for each subsequent year of the 20 year time horizon since the baby is assumed to die (ie, years lost from a child's life were valued the same as years lost from an adult's life). We assumed no monetary costs as a result of neural tube defects, except in a sensitivity analysis. Depending on an HIV-positive woman's viral load, birth of an HIV-infected child can occur. We assumed that an HIV-infected child will access ART and that an additional 0·1 DALYs, and cost of $160 per year are incurred for each subsequent year of the 20 year time horizon.

### Role of the funding source

The funder of the study had no role in study design, modelling approach, interpretation, or writing of the report. The corresponding author had full access to all model programs and outputs in the study and had final responsibility for the decision to submit for publication.

## Results

Consequences of the various regimen policies for the use of each drug are shown in table 3. A mean of 98% of people receiving ART over 20 years would be expected to be on dolutegravir with the policy of tenofovir, lamivudine, and dolutegravir for all, compared with only 43% if its use was dependent on viral load suppression and being male or a woman not intending to have (more) children.

**Table 3:** Predicted effect of regimen policies on intermediate outcomes, DALYs, and net DALYs

		**Tenofovir, lamivudine, and efavirenz for all**	**Tenofovir, lamivudine, and dolutegravir dependent on viral suppression and intention to have (more) children**	**Tenofovir, lamivudine, and dolutegravir dependent on intention to have (more) children only**	**Tenofovir, lamivudine, and dolutegravir dependent on viral suppression only**	**Tenofovir, lamivudine, and dolutegravir for all**
Proportion on efavirenz		92%	52%;–40% (−41 to −39; −52 to −22)	42%; −50% (−51% to −49; −61 to −39)	15%; −77% (−78 to −76; −92 to −47)	0%; −92% (−93 to −91; −100 to −78)
Proportion on dolutegravir		0%	43%; 43% (42 to 44; 22 to 57)	54%; 54% (53 to 55; 42 to 63)	85%; 83% (82 to 85; 47 to 97)	98%; 98% (97 to 99; 94 to 100)
Proportion on atazanavir		8%	5%; −3% (−4 to −2; −9 to 0)	4%; −3% (−4 to −2; −9 to −1)	2%; −6% (−7 to −5; −16% to 0)	2%; −6% (−7 to −5; −16 to 0)
Proportion on zidovudine*		8%	5%; −2% (−3 to −1; −8 to 0)	4%; −4% (−5 to −3; −10 to −2)	4%; −4% (−5 to −3; −13 to 0)	2%; −6% (−7 to −5; −16 to 0)
Proportion of people on ART with viral load <1000 copies per mL						
	Mean over 1 year	84%	85%; 0% (0 to 0; 0 to 2)	87%; 2% (2 to 2; 0 to 5)	85%; 1% (1 to 1; −1 to 3)	90%; 6% (6 to 6; 1 to 11)
	Mean over 5 years	84%	86%; 2% (2 to 2; 0 to 4)	87%; 3% (3 to 3; 0 to 7)	88%; 4% (4 to 4; 1 to 8)	91%; 7% (7 to 7; 1 to 14)
	Mean over 20 years	82%	87%; 5% (5 to 5; 1 to 9)	87%; 5% (5 to 5; 1 to 11)	91%; 9% (9 to 9; 3 to 16)	91%; 10% (10 to 10; 2 to 18)
Of people with baseline viral load >1000 copies per mL and Lys65Arg and Met184Val mutations at baseline, percentage of people on ART with viral load <1000 copies per mL						
	Mean over 1 year	7%	9%; 3% (2 to 4; −1 to 8)	23%; 16% (15 to 17; 4 to 28)	14%; 7% (6 to 8; −1 to 21)	49%; 42% (40 to 44; 15 to 64)
	Mean over 5 years	22%	26%; 4% (3 to 5%; −1 to 10)	35%; 13% (12 to 14; −3 to 32)	33%; 11% (9 to 13; −1 to 25)	55%; 32% (29 to 35; −3 to 72%)
	Mean over 20 years	50%	50%; 0% (−1 to 1; −9 to 8)	52%; 2% (−1 to 5; −16 to 46)	53%; 3% (1 to 5; −15 to 18)	57%; 7% (3 to 11; −22 to 64)
AIDS death rate in people on ART (per 100 person-years)		1·70	1·25; −0·46 (−0·48 to −0·44; −0·94 to −0·12)	1·08; −0·63 (−0·66 to −0·60; −1·34 to −0·14)	0·94; −0·76 (−0·79 to −0·73; −1·51 to −0·24)	0·72; −0·98 (−1·02 to −0·94; −2·02 to −0·24)
AIDS death rate in people on ART with viral load >1000 copies per mL (per 100 person-years)		7·05	6·29; −0·76 (−0·82 to −0·70; −1·41 to −0·12)	5·71; −1·34 (−1·44 to −1·24; −2·54 to −0·45)	5·92; −1·12 (−1·22 to −1·02; −2·22 to −0·14)	4·77; −2·28 (−2·08 to −2·48; −4·36 to −0·48)
AIDS death rate in people on ART with viral load >1000 copies per mL and CD4 count <200 cells per μL (per 100 person-years)		18·07	17·22; −0·85 (−1·24 to −0·66; −2·01 to 0·91)	16·05; −2·02 (−2·26 to 1·78; −4·15 to −0·11)	17·05; −1·03 (−1·34 to −0·72; −3·41 to 1·45)	14·77; −3·30 (−3·78 to −2·82; −8·43 to 0·03)
Proportion of all HIV-positive people with a dolutegravir resistance mutation		0%	2·6%; 2·6% (2·3 to 2·7; 0·4 to 7·7)	4·0%; 4·0% (3·8 to 4·2; 0·6 to 11·0)	4·4%; 4·4% (4·1 to 4·7; 0·7 to 12·9)	6·7%; 6·7% (6·2 to 7·0; 1·2 to 18·5)
Proportion of all HIV-positive people with an efavirenz resistance mutation		28%	22%; −6% (−6 to −6; −12 to −1)	20%; −8% (−8 to −8; −14 to −3)	15%; −13% (−13 to −13; −22 to −6)	13%; −15% (−15 to 15; −24 to −8)
Adverse birth outcomes among women with HIV (percentage of pregnancies)						
	Mother-to-child transmission	4·2%	3·9%; −0·2% (−0·2 to −0·2; −0·8 to 0·3)	3·8%; −0·3% (−0·3 to −0·3; −1·1 to 0·3)	2·9%; −1·2% (−1·2 to −1·2; −2·5 to −0·3)	2·8%; −1·4 (−1·4 to −1·4; −2·9 to −0·3)
	Neural tube defect due to dolutegravir†	0%	0·02%; 0·02% (0·02 to 0·02; 0·0 to 0·08)	0·03%; 0·03% (0·03 to 0·03; 0·0 to 0·09%)	0·52%; 0·52% (0·49 to 0·55; −0·12 to 1·38)	0·60%; 0·60% (0·58 to 0·62; 0·15 to 1·51)
Costs‡ (annual difference compared with tenofovir, lamivudine, and efavirenz)§		..	−$5·3 million (−$5·8 million to −$4·8 million; −$19·6 million to $2·0 million)	−$5·3 million (−$4·8 million to −$5·8 million; −$20·4 million to $3·1 million)	−$10·5 million (−$11·3 million to $9·7 million; −$37·4 million to $1·4 million)	−$9·7 million (−$10·6 million to −$8·8 million; −$38·0 million to $3·9 million)
DALYs averted‡ (per year, compared with tenofovir, lamivudine, and efavirenz)¶		..	22 300 (21 300 to 23 300; 1400 to 53 000)	32 800 (31 300 to 34 300; 3900 to 78 200)	39 500 (37 000 to 42 000; 7900 to 87 900)	58 200 (55 700 to 61 300; 11 500 to 138 300)
Incremental cost-effectiveness ratio‖		Dominated	Dominated	Dominated	Reference	$44
Net DALYs (per year, compared with tenofovir, lamivudine, and efavirenz)¶		..	32 900 (31 400 to 34 400; 2700 to 84 200)	43 500 (41 700 to 45 300; 8600 to 103 100)	60 600 (58 000 to 63 200; 11 800 to 143 400)	77 700 (74 700 to 80 700; 20 800 to 177 600)

Data are mean proportion and mean difference (95% CI; 90% range reflecting variation across setting scenarios) compared with the policy of tenofovir, lamivudine, and efavirenz for all, unless otherwise indicated. DALYs and net DALYs were measured over 20 years (2018–38) and represent the mean over 3 month periods, unless otherwise stated. Costs are in US$. ART=antiretrovoral therapy. DALY=disability adjusted life-year.

* As opposed to tenofovir disoproxil fumarate, all are on lamivudine.

† Neural tube defects potentially caused by dolutegravir if possible signal is confirmed.

‡ In the context of an adult population of 10 million people with HIV prevalence as in table 2. Costs and DALYs are discounted at 3% per annum.

§ Values are mean and mean difference (95% CI; 90% range reflecting variation across setting scenarios).

¶ Values are n per year (95% CI; 90% range reflecting variation across setting scenarios).

‖ Policies are dominated if there is another policy with both lower cost and more DALYs averted. The reference policy is selected among non-dominated policies.

The predicted effect on viral load suppression is shown in people on ART over a mean of 1 year, 5 years, and 20 years (table 3). Restriction of tenofovir, lamivudine, and dolutegravir use in women who intend to have (more) children was predicted to lead to poorer overall viral suppression over 1 year, 5 year, and 20 year time periods. Dependence of use of tenofovir, lamivudine, and dolutegravir on viral load suppression was predicted to lead to reduced overall viral suppression over 1 year and 5 year time periods, although we found little difference in viral suppression over the 20 year time period. Restriction of analysis to the subgroup of people with viral loads greater than 1000 copies per mL and presence of drug mutations to tenofovir and lamivudine at baseline revealed a markedly higher predicted proportion of people with viral load suppression over the 1 year time period for the policy of tenofovir, lamivudine, and dolutegravir for all compared with the other policies, because with this policy a switch was made to tenofovir, lamivudine, and dolutegravir and with the dependence on viral suppression policies no switch was made. This difference persisted with longer follow-up durations but was less marked because of identification of virological failure and switching to zidovudine, lamivudine, and dolutegravir or zidovudine, lamivudine, and a protease inhibitor occurs with the policy of tenofovir, lamivudine, and dolutegravir dependent on viral suppression. Since this subgroup has a tendency for poorer adherence, and with our assumptions about resistance to dolutegravir and the residual activity of tenofovir and lamivudine, the percentage of patients with viral suppression was predicted to remain at less than 60% with all policies, including tenofovir, lamivudine, and dolutegravir for all. Use of tenofovir, lamivudine, and dolutegravir was predicted to lead to more future dolutegravir resistance compared with tenofovir, lamivudine, and dolutegravir dependent on viral suppression (mean 6·7%, 90% range across setting scenarios 1·2–18·5 *vs* 4·4%, 0·7–12·9% in the context of no dependence on intention to have more children; table 3). In the final year of the 20 year time horizon, the corresponding figures were 9·4% (1·6–26·3) and 7·6% (1·4–21·3; data not shown).

The number of deaths due to AIDS in people on ART showed a similar pattern to overall viral suppression over the 1 year and 5 year time horizons, with AIDS deaths declining with increasing use of tenofovir, lamivudine, and dolutegravir, and at their lowest for the policy of tenofovir, lamivudine, and dolutegravir for all (table 3). Differences between policies in people on ART with viral load suppression translate into substantial differences in AIDS deaths because of the high death rate in those without viral suppression at any point in time. The lower death rate in those without viral suppression in the tenofovir, lamivudine, and dolutegravir for all policy is due to a lower CD4 count, even within the CD4 count less than 200 cells per μL range.

The predicted proportion of HIV-positive women for whom mother-to-child transmission occurs also followed this pattern. The reduction in risk of mother-to-child transmission with the tenofovir, lamivudine, and dolutegravir for all policy was greater in absolute terms than the increased risk of neural tube defects with the same policy (table 3).

There were substantially more DALYs averted with the tenofovir, lamivudine, and dolutegravir for all policy than for any of the policies in which tenofovir, lamivudine, and dolutegravir use was dependent on viral suppression or intention to have no (more) children. The lowest cost was for the tenofovir, lamivudine, and dolutegravir for all and tenofovir, lamivudine, and dolutegravir dependent on viral suppression only policies (figure). For net DALYs, the benefit of the tenofovir, lamivudine, and dolutegravir for all policy was greater still, reflecting the health gains elsewhere due to the lower costs, mainly because of reduced use of protease inhibitors, which are more than 7 times the cost of dolutegravir. The policy of tenofovir, lamivudine, and dolutegravir for all was the most cost-effective in 83% of setting scenarios, with tenofovir, lamivudine, and dolutegravir dependent on viral suppression only the most cost-effective policy in 16% of setting scenarios. Tenofovir, lamivudine, and dolutegravir dependent on intention to have (more) children only was cost-effective in 1% of setting scenarios and tenofovir, lamivudine, and dolutegravir dependent on viral suppression and intention to have (more) children was cost-effective in less than 1% of setting scenarios (table 4). Tenofovir, lamivudine, and efavirenz for all was not cost-effective in any setting scenario.

**Figure:**
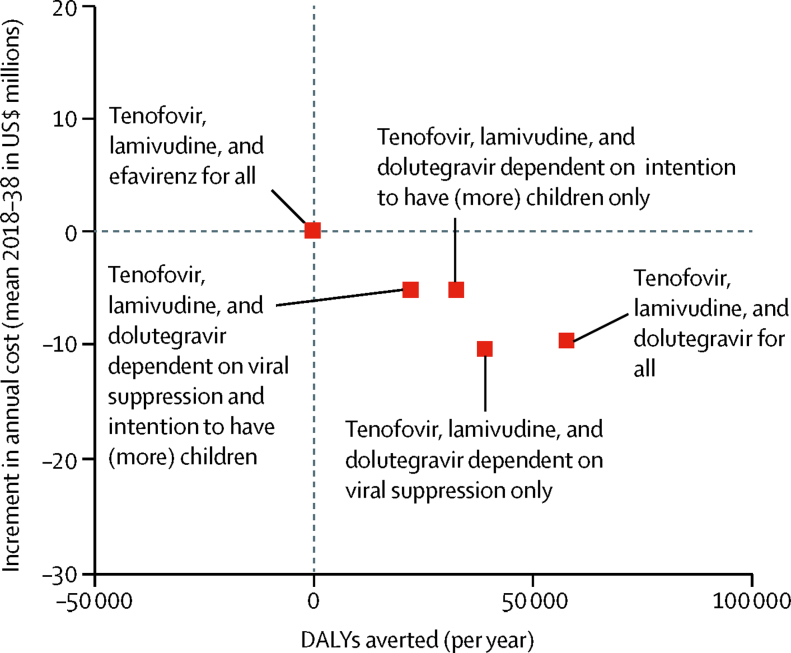
Overall health benefit (DALYs averted) and increment in cost compared with tenofovir, lamivudine, and efavirenz for all Values are the mean over 3 month periods of 1000 setting scenarios during 20 year time periods, expressed per year. DALY=disability adjusted life-year.

**Table 4:** Sensitivity analyses

		**DALYs averted**				Net DALYs averted (% of setting scenarios in which policy is the most cost-effective*)			
		Tenofovir, lamivudine, and dolutegravir dependent on viral suppression and intention to have (more) children	Tenofovir, lamivudine, and dolutegravir dependent on intention to have (more) children only	Tenofovir, lamivudine, and dolutegravir dependent on viral suppression only	Tenofovir, lamivudine, and dolutegravir for all	Tenofovir, lamivudine, and dolutegravir dependent on viral suppression and intention to have (more) children	Tenofovir, lamivudine, and dolutegravir dependent on intention to have (more) children only	Tenofovir, lamivudine, and dolutegravir dependent on viral suppression only	Tenofovir, lamivudine, and dolutegravir for all
No restriction (base case)		22 300 (21 300 to 23 300; 1400 to 53 000)	32 800 (31 300 to 34 300; 3900 to 78 200)	39 500 (37 000 to 42 000; 7900 to 87 900)	58 200 (55 700 to 61 300; 11 500 to 138 300)	32 900 (<1%) (31 400 to 34 400; 2700 to 84 200)	43 500 (1%) (41 700 to 45 300; 8600 to 103 100)	60 600 (16%) (58 000 to 63 200; 11 800 to 143 400)	77 700 (83%) (74 700 to 80 700; 20 800 to 177 600)
Restrictions to setting scenarios									
	Development of resistance to dolutegravir is 3 times higher	20 300 (18 100 to 22 500; 0 to 50 400)	29 100 (25 800 to 32 400; 3000 to 72 800)	35 800 (32 400 to 39 200; 8900 to 81 400)	50 100 (44 700 to 55 500; 4800 to 124 500)	29 700 (0%) (26 700 to 32 700; 3400 to 72 800)	38 300 (1%) (34 700 to 41 900; 9200 to 88 800)	54 400 (24%) (49 400 to 59 400; 11 900 to 127 200)	66 600 (75%) (50 900 to 72300; 21 000 to 149 100)
	Toxicity to dolutegravir the same as efavirenz	21 800 (19 600 to 24000; 2800 to 52 300)	31 600 (28 400 to 34 800; 3200 to 73 500)	38 800 (35 200 to 42 400; 6600 to 87 900)	58 400 (52 800 to 64 000; 11 800 to 131 100)	32 100 (1%) (28 600 to 35 600; 3000 to 84 100)	41 800 (1%) (37 800 to 45 800; 9300 to 91 700)	59 100 (16%) (53 300 to 64 900; 9800 to 141 900)	76 600 (82%) (69 900 to 83 300; 19 500 to 167 400)
	Potency of dolutegravir 1·0 (equal to efavirenz)	16 900 (15 200 to 18 600; −1000 to 40 600)	23 700 (21 100 to 26 300; −100 to 53 900)	30 200 (27 500 to 32 900; 4400 to 62 900)	41 900 (37 800 to 46 000; 2200 to 93 900)	24 000 (1%) (21 500 to 26 500; 900 to 52 500)	30 900 (1%) (28 100 to 33 700; 6500 to 63 600)	45 400 (31%) (41 300 to 49 500; 9600 to 95 000)	54 600 (68%) (50 200 to 59 000; 17 700 to 108 800
	Potency of dolutegravir 1·0 (equal to efavirenz) plus development of resistance to dolutegravir 3 times higher	13 700 (10 300 to 17 100; −2600 to 33 500)	18 600 (13 700 to 23 500; −1300 to 53 800)	25 300 (19 800 to 30 800; 4000 to 57 100)	32 000 (23 600 to 40 400; 1000 to 91 000)	19 100 (0%) (15 100 to 23 100; −1300 to 41 000)	23 100 (3%) (18 700 to 27 500; 3300 to 47 700)	35 600 (49%) (29 800 to 41 400; 9200 to 72 000)	39 100 (49%) (31 900 to 46 300; 4900 to 84 500)
	Pre-ART non-nucleoside reverse-transcriptase inhibitor resistance in 2018 >10%	27 900 (26 40 to 29 400; 5100 to 61 400)	40 400 (38 100 to 42 700; 8600 to 93 800)	48 800 (46 300 to 51 300; 12 800 to 104 700)	71 100 (67 700 to 75 100; 19 300 to 157 300)	41 500 (0%) (39 100 to 43 900; 7300 to 97 200)	54 200 (<1%) (51 500 to 56 900; 14 200 to 118 000)	76 100 (14%) (72 100 to 80 100; 21 700 to 172 000)	96 400 (86%) (92 000 to 100 800; 34 500 to 20 300)
	Pre-ART non-nucleoside reverse-transcriptase inhibitor resistance in 2018 <5%	12 700 (11 200 to 14 200; −2500 to 34 400)	20 100 (17 900 to 22 300; −300 to 51 900)	23 400 (21 300 to 23 500; 1500 to 51 100)	36 100 (32 400 to 39 800; 3800 to 87 000)	18 200 (1%) (16 200 to 20 200; 300 to 42 700)	25 600 (2%) (23 100 to 28 100; 2800 to 59 400)	34 600 (21%) (31 500 to 37 700; 6000 to 72 200)	46 400 (76%) (42 500 to 50 300; 13 700 to 106 700)
	Zero residual activity of tenofovir and lamivudine in the presence of Lys65Arg or Met184Val mutations	26 600 (20 900 to 32 300; 2500 to 67 900)	35 300 (27 300 to 43 300; 5300 to 94 800)	46 700 (37 500 to 55 900; 6300 to 109 500)	62 700 (49 400 to 76 000; 10 500 to 168 200)	37 600 (0%) (29 400 to 45 800; 2500 to 98 500)	45 000 (0%) (35 800 to 54 200; 5800 to 108 500)	68 300 (26%) (54 300 to 82 300; 18 400 to 172 100)	80 500 (74%) (64 800 to 96 200; 20 290 to 198 394)
	Rate of switch after virological failure 0·05 per 3 months	25 000 (23 000 to 27 000; 1700 to 57 900)	37 200 (34 300 to 40 100; 5400 to 85 600)	44 900 (41 700 to 48 100; 10 400 to 101 700)	65 700 (61 100 to 70 300; 18 300 to 145 000)	31 400 (<1%) (28 600 to 34 200; 300 to 78 500)	42 800 (1%) (39 500 to 46 100; 6500 to 103 600)	58 500 (14%) (54 000 to 63 000; 12 400 to 135 800)	76 500 (84%) (71 100 to 81 900; 19 500 to 178 100)
	Rate of switch after virological failure 0·5 per 3 months	17 100 (15 400 to 19 800; −600 to 38 700)	26 300 (23 700 to 28 900; 300 to 66 200)	31 500 (28 700 to 34 300; 4400 to 63 300)	46 900 (41 400 to 51 400; 3800 to 111 200)	28 900 (<1%) (26 200 to 31 600; 3100 to 72 700)	38 900 (1%) 35 800 to 42 000; 8800 to 81 800	54 400 (18%) (39 500 to 58 900; 11 600 to 132 400)	69 200 (81%) (64 100 to 74 300; 22 600 to 143 500
	Proportion of women giving birth per 3 month period >4%	24 700 (22 000 to 27 400; 2300 to 53 400)	36 600 (32 500 to 40 700; 2200 to 95 600)	43 500 (41 300 to 47 700; 12 700 to 91 200)	65 800 (59 000 to 72 600; 11 900 to 160 700)	35 000 (1%) (31 000 to 39 000; 4900 to 89 700)	47 000 (0%) (42 400 to 51 600; 11 500 to 106 400)	64 800 (11%) (58 400 to 71 200; 18 000 to 144 200)	85 400 (89%); 77 600 to 93 200; 19 800 to 184 000)
	Proportion of women giving birth per 3 month period <4%	21 900 (20 800 to 23 000; 1400 to 52 600)	32 100 (30 400 to 33 800; 3900 to 75 900)	38 800 (37 000 to 40 600; 7000 to 87 800)	56 700 (54 000 to 59 400; 11 308 to 131 100)	32 500 (<1%) (30 700 to 34 300; 2200 to 84 100)	42 800 (1%) (40 800 to 44 800; 7800 to 97 100)	59 800 (17%) (56 900 to 63 700; 10 800 to 142 600)	76 100 (82%) (72 900 to 79 300; 20 900 to 168 000)
	Risk of neural tube defects with dolutegravir 1·0%	22 300 (21 300 to 23 300; 1400 to 52 800)	32 800 (31 300 to 34 300; 3900 to 78 200)	38 600 (37 000 to 40 200; 7500 to 86 900	57 100 (54 600 to 59 600; 10 600 to 136 100)	32 900 (<1%) (31 300 to 34 500; 2700 to 84 200)	43 400 (1%) (41 600 to 45 200; 8600 to 102 900)	59 700 (16%) (57 100 to 62 300; 11 600 to 141 700)	76 500 (84%) (75 000 to 78 000; 20 300 to 175 500)
	Risk of neural tube defects with dolutegravir 3·0%	22 100 (21 100 to 23 100; 1400 to 52 700)	32 500 (31 000 to 34 000; 3700 to 78 200)	34 300 (32 800 to 35 800; 4300 to 81 300)	51 600 (49 100 to 54 100; 5900 to 127 900)	32 700 (<1%) (31 200 to 34 200; 2500 to 84 100)	43 200 (1%) (41 400 to 45 400; 8600 to 102 000)	55 400 (16%) (52 900 to 57 900; 9700 to 132 700)	71 000 (82%) (68 200 to 73 800; 18 000 to 166 800)
	Viral load testing fully implemented	19 100 (17 300 to 20 900; 800 to 43 000)	21 000 (18 900 to 23 100; −1700 to 48 500)	32 800 (30 000 to 35 600; 6700 to 66 800)	36 200 (32 800 to 39 600; 2500 to 77 500)	40 700 (0%) (37 200 to 44 200; 8400 to 92 300)	44 100 (2%) (40 300 to 47 900; 9600 to 98 200)	72 800 (28%) (66 900 to 78 700; 23 600 to 150 500)	78 200 (70%) (71 900 to 84 500; 23 900 to 173 600)
	Viral load testing not implemented	19 100 (17 200 to 21 000; −2500 to 44 400)	43 800 (40 100 to 47 000; 7700 to 96 500)	34 800 (31 800 to 37 800; 2900 to 76 100)	79 300 (73 400 to 85 200; 21 600 to 159 100)	18 300 (0%) (16 300 to 20 300; −2800 to 44 600)	41 400 (0%) (37 800 to 45 000; 5600 to 88 500)	35 700 (3%) (32 500 to 37 900; 1200 to 82 500)	75 000 (98%) (69 200 to 80 800; 18 300 to 152 800)
	Higher background treatment interruption	20 900 (18 900 to 22 900; −300 to 51 300)	29 900 (27 100 to 32 700; 3000 to 76 400)	39 000 (35 900 to 42 100; 10 200 to 89 100)	56 100 (51 500 to 60 700; 15 700 to 128 000	27 100 (0%) (24 300 to 29 900; 500 to 72 900)	36 100 (1%) (32 800 to 39 400; 3500 86 400)	52 300 (16%) (47 700 to 56 900; 9800 to 127 700)	67 500 (83%) (62 000 to 73 000; 16 700 to 151 200)
	HIV prevalence (age 15–49 years) in 2018 <8%	12 700 (11 500 to 13 900; −1900 to 29 100)	19 200 (17 700 to 20 700; 3000 to 40 500)	22 800 (21 100 to 24 500; 2000 to 50 800)	33 500 (31 200 to 35 800; 7000 to 65 400)	17 900 (1%) (16 200 to 19 600; −1300 to 40 100)	24 400 (2%) (22 600 to 26 200; 3500 to 52 100)	32 900 (22%) (30 300 to 35 500; 3200 to 69 000)	42 600 (76%) (40 000 to 45 200; 14 000 to 81 100)
	HIV prevalence (age 15–49 years) in 2018 >12%	32 200 (30 200 to 34 200; 7900 to 68 400)	47 100 (44 100 to 50 100; 10 800 to 101 400)	56 300 (53 300 to 59 300; 17 100 to 114 000)	83 700 (78 800 to 88 600; 23 000 to 171 900)	48 400 (0%) (45 400 to 51 400; 12 000 to 103 000)	63 400 (<1%) (60 100 to 66 700; 21 900 to 121 200)	89 300 (10%) (84 400 to 94 200; 30 100 to 184 100)	114 500 (90%) (109 300 to 119 700; 45 900 to 209 200)
	HIV incidence (age 15–49 years) in 2018 (per 100 person-years) <0·6	12 800 (11 600 to 14 000; −1600 to 28 600)	20 000 (18 200 to 21 800; 1700 to 43 800)	22 000 (20 300 to 23 700; 2900 to 45 700)	34 400 (31 700 to 37 100; 6900 to 69 000)	19 400 (0%) (17 600 to 21 200; −1000 to 42 100	26 600 (<1%) (24 600 to 28 600; 6400 to 55 200)	33 900 (20%) (31 200 to 36 600; 3200 to 67 200)	45 300 (78%) (42 300 to 48 300; 17 600 to 83 900)
	HIV incidence (age 15–49 years) in 2018 (per 100 person-years) >1	30 800 (28 800 to 32 800; 5100 to 68 400)	44 600 (41 600 to 47 600; 8600 to 99 500)	55 400 (52 300 to 58 500; 16 600 to 114 000)	81 000 (76 200 to 85 800; 21 900 to 167 500)	45 000 (0%) (42 000 to 48 000; 8300 to 100 200)	58 900 (0%) (55 500 to 62 300; 13 800 to 121 000)	84 900 (12%) (79 900 to 89 900; 26 800 to 181 900)	108 000 (88%) (102 500 to 113 500; 36 500 to 208 500)
	Proportion of HIV-positive people diagnosed in 2018 <75%	17 700 (15 600 to 19 800; 200 to 41 500)	25 200 (22 400 to 28 000; 3000 to 56 200)	36 200 (32 700 to 39 700; 6300 to 74 200)	49 800 (44 800 to 54 800; 11 900 to 111 900)	22 900 (0%) (20 000 to 25 800; 500 to 58 400)	30 300 (1%) (26 900 to 33 700; 3900 to 68 300)	47 900 (21%) (42 400 to 52 900; 8200 to 120 000)	59 900 (78%) (54 100 to 65 700; 17 600 to 130 200)
	Proportion of HIV positive people diagnosed in 2018 >88%	25 400 (23 300 to 27 500; 2000 to 59 900)	37 800 (34 500 to 41 100; 5000 to 93 800)	42 300 (38 900 to 45 700; 8300 to 103 700)	64 700 (61 400 to 70 000; 16 000 to 152 000)	39 700 (0%) (36 100 to 43 300; 5300 to 98 400)	52 000 (0%) (48 100 to 55 900; 10 400 to 118 900)	68 400 (11%) (62 700 to 74 100; 11 100 to 162 200)	88 900 (89%) (82 600 to 95 200; 25 900 to 200 500)
	Proportion of ART-experienced people who started second-line (boosted protease inhibitor) ART in 2018 <1·5%	19 900 (17 900 to 21 900; −300 to 47 000)	34 200 (31 300 to 37 100; 5000 to 75 900)	38 200 (35 200 to 41 200; 8200 to 82 900)	63 300 (58 700 to 67 900; 16 700 to 134 900)	21 300 (<1%) (19 100 to 23 500; −1500 to 55 900)	34 700 (1%) (31 700 to 37 700; 2600 to 79 600)	42 700 (11%) (39 000 to 46 400; 6200 to 101 800)	64 300 (88%) (59 500 to 70 100; 14 900 to 136 300)
	Proportion of ART-experienced people who started second-line (boosted protease inhibitor) ART in 2018 >4%	20 600 (18 800 to 22 400; 1200 to 47 000)	23 900 (21 700 to 26 100; −1300 to 54 600)	34 800 (32 100 to 36 500; 6400 to 75 500)	39 600 (36 100 to 43 100; 3200 to 93 600)	44 900 (0%) (41 700 to 48 100; 12 100 to 98 800)	49 800 (1%) (46 300 to 53 300; 11 600 to 107 700)	80 100 (27%) (74 800 to 85 400; 26 100 to 162 700)	86 500 (72%) (82 600 to 92 400; 26 800 to 185 500)
	Of people on ART, proportion with viral load <1000 copies per mL in 2018 <80%	32 800 (29 600 to 36 000; 10 400 to 74 000)	48 400 (43 600 to 53 200; 10 700 to 109 500)	59 000 (54 000 to 64 000; 17 400 to 129 500)	87 300 (84 800 to 89 800; 24 300 to 185 900)	40 900 (0%) (36 400 to 45 400; 3800 to 98 500)	55 400 (0%) (50 000 to 60 800; 7400 to 122 900)	77 500 (11%) (70 900 to 87 100; 18 200 to 185 700)	101 400 (89%) (98 400 to 104 400; 31 000 to 218 800)
	Of people on ART, proportion with viral load <1000 copies per mL in 2018 >88%	13 700 (12 400 to 15 000; −1600 to 30 900)	17 600 (15 800 to 19 400; −1800 to 40 600)	23 600 (21 600 to 25 600; 1900 to 51 100)	30 000 (27 200 to 32 800; 1300 to 67 800)	27 300 (1%) (24 700 to 29 900; 2200 to 61 300)	32 200 (2%) (29 500 to 34 900; 6800 to 69 100)	48 900 (26%) (44 800 to 53 000; 11 000 to 111 200)	56 100 (71%) (51 800 to 60 400; 14 500 to 120 100)
	Cost-effectiveness threshold US$200	22 300 (21 300 to 23 300; 1400 to 53 000)	32 800 (31 300 to 34 300; 3900 to 78 200)	39 500 (37 000 to 42 000; 7900 to 87 900)	58 200 (55 700 to 61 300; 11 500 to 138 300)	48 700 (<1%) (45 900 to 51 500; −100 to 107 200)	59 400 (1%) (56 500 to 62 300; 8400 to 156 900)	92 300 (21%) (87 300 to 97 300; 12 600 to 252 200)	106 900 (78%) (101 900 to 111 900; 20 700 to 270 100)
	Cost-effectiveness threshold US$1000	22 300 (21 300 to 23 300; 1400 to 53 000)	32 800 (31 300 to 34 300; 3900 to 78 200)	39 500 (37 000 to 42 000; 7900 to 87 900)	58 200 (55 700 to 61 300; 11 500 to 138 300)	27 600 (<1%) 26 300 to 28 900; 3100 to 66 000	38 200 (1%) (36 600 to 39 800; 7200 to 87 300)	50 100 (14%) (48 100 to 52 100; 12 600 to 111 600)	67 900 (85%) (65 300 to 70 500; 18 800 to 153 100)
Additional sensitivity analyses									
	Dolutegravir potency becomes 0·75 during tuberculosis treatment	21 700 (18 700 to 24 700; 4800 to 42 900)	31 300 (26 300 to 36 300; 2700 to 69 400)	38 600 (33 800 to 43 400; 6700 to 73 700)	55 300 (47 100 to 63 500; 13 500 to 122 600)	31 800 (0%) (27 500 to 36 100; 5600 to 72 400)	41 500 (0%) (36 300 to 46 700; 15 200 to 85 700)	58·700 (15%) (51 800 to 65 600; 16 200 to 134 900)	74 900 (85%) (68 300 to 83 500; 23 500 to 158 700)
	For policies with dependence on viral suppression, people on zidovudine, lamivudine, and protease inhibitor (atazanavir) are moved to zidovudine, lamivudine, and dolutegravir rather than tenofovir, lamivudine, and dolutegravir	23 100 (20 800 to 25 400; 1300 to 52 800)	33 000 (29 600 to 36 400; 6200 to 68 100)	40 800 (37 100 to 44 500; 10 400 to 83 700)	56 800 (53 400 to 60 200; 13 000 to 131 700)	33 900 (0%) (30 500 to 37 300; 3900 to 81 200)	43 700 (0%) (39 900 to 47 500; 11 400 to 90 000)	62 300 (20%) (66 600 to 68 000; 14 400 to 128 900)	76 900 (80%) (70 400 to 83 400; 22 500 to 159 100)
	Higher mother-to-child transmission	20 000 (18 000 to 22 000; 1100 to 46 300)	29 700 (26 700 to 32 700; 2600 to 75 800)	36 400 (33 400 to 39 400; 5900 to 80 000)	54 000 (49 200 to 54 800; 9100 to 123 900)	30 800 (0%) (27 900 to 33 700; 4500 to 68 900)	40 700 (0%) (37 400 to 44 000; 9000 to 85 800)	58 300 (15%) (53 600 to 63 000; 14 300 to 119 800)	75 600 (85%) (70 100 to 81 100; 23 600 to 160 800)
	Defects not fatal in 50% of babies born with neural tube defects and lifetime disability weight and cost incurred for surviving babies†	21 100 (18 900 to 23 300; 1400 to 50 200)	29 900 (26 800 to 33 000; 2000 to 67 500)	37 800 (34 400 to 41 200; 8800 to 79 600)	53 900 (48 800 to 59 000; 10 000 to 112 700)	32 900 (5%) (29 700 to 36 100; 2500 to 73 300)	42 100 (18%) (38 500 to 45 700; 6500 to 88 600)	60 900 (14%) (55 800 to 66 000; 13 000 to 131 700)	76 000 (63%) (70 200 to 81 800; 22 100 to 162 300)
	Contraception has 50% effectiveness instead of 80%	24 000 (21 300 to 26 700; 2600 to 54 900)	36 900 (33 000 to 40 800; 2700 to 87 700)	41 500 (37 500 to 45 500; 9400 to 89 600)	64 500 (58 500 to 71 100; 8400 to 142 700)	34 700 (1%) (30 700 to 38 700; 5700 to 83 500)	47 300 (1%) (43 800 to 51 800; 9600 to 105 800)	62 300 (19%) (56 000 to 68 600; 14 700 to 137 900)	83 200 (80%) (75 800 to 90 600; 19 300 to 178 400)
	Efavirenz has potency 1·5	20 200 (17 400 to 23 000; −500 to 50 500)	28 300 (24 100 to 32 400; 0 to 84 600)	35 300 (30 500 to 40 100; 2700 to 91 000)	50 600 (53 900 to 57 300; 6800 to 132 200)	29 600 (1%) (25 600 to 33 600; 4100 to 73 000)	38 100 (2%) (33 100 to 43 100; 5100 to 98 900)	54 000 (15%) (47 300 to 60 700; 12 800 to 133 700)	68 000 (82%) (60 400 to 75 600; 19 200 to 158 500)
	Viral load threshold 50 copies per mL	18 100 (15 000 to 21 200; −5200 to 48 600)	28 800 (24 400 to 33 200; 1200 to 69 000)	30 000 (25 800 to 34 200; 4800 to 66 600)	50 000 (43 100 to 56 900; 4800 to 108 000)	28 800 (0%) (24 100 to 33 500; −700 to 67 200)	40 300 (6%) (34 900 to 45 700; 6800 to 80 800)	50 900 (9%) (43 800 to 58 000; 6700 to 106 800)	68 100 (86%) (59 700 to 76 500; 16 000 to 139 200)
	Atazanavir resistance rate 3 times lower	22 600 (18 600 to 26 600; 4200 to 60 800)	33 100 (26 600 to 39 600; 7500 to 85 700)	37 500 (31 900 to 43 300; 7300 to 86 400)	56 600 (46 400 to 66 800; 17 000 to 128 000)	34 600 (0%) (28 200 to 41 000; 6600 to 78 900)	45 700 (2%) (38 100 to 53 300; 12 200 to 107 300)	60 400 (15%) (49 800 to 71 000; 13 100 to 130 300)	78 200 (83%) (66 200 to 90 200; 23 900 to 150 000)
	50 year time horizon	49 000 (41 800 to 56 200; 700 to 136 000)	60 000 (50 800 to 69 200; 15 800 to 178 800)	84 200 (72 800 to 95 600; 9800 to 218 700)	93 200 (79 500 to 106 900; 8800 to 269 300)	57 800 (0%) (50 400 to 65 200; 1800 to 127 900)	69 100 (0%) (60 600 to 77 600; 4200 to 161 500)	99 800 (0%) (88 600 to 111 000; 15 600 to 211 200)	107 200 (100%) (94 600 to 119 800; 12 600 to 242 200)

Values are DALYs and net DALYs averted compared with the policy of tenofovir, lamivudine, and efavirenz for all (95% CI; 90% range, reflecting variation across setting scenarios), unless otherwise indicated. DALY=disability adjusted life-year. ART=antiretroviral therapy.

* Tenofovir, lamivudine, and efavirenz for all policy was the most cost-effective policy in 0% of all setting scenarios.

† Disability weight for child living with neural tube defect is 0·5, cost is US$5000 in the first year (cost of surgery) and $1000 per year (cost of ongoing care and support) thereafter.

Tenofovir, lamivudine, and dolutegravir for all remained the most effective and cost-effective policy in several sensitivity analyses (table 4).

## Discussion

Supporting earlier work,^31^ we found that the benefits of a policy using tenofovir, lamivudine, and dolutegravir in people currently on first-line or second-line regimens, without any dependence on viral load or consideration of use of zidovudine rather than tenofovir, outweighed the risks. We also found that there were substantial net public health benefits of use of tenofovir, lamivudine, and dolutegravir, including in women of child bearing age, in terms of DALYs incurred in the whole population, and considering DALYs incurred as a result of birth of a child with a neural tube defect.

The benefits of the policy of tenofovir, lamivudine, and dolutegravir for all compared with tenofovir, lamivudine, and dolutegravir dependent on viral suppression are due to proactive use of dolutegravir without requiring a viral load measure or a switch algorithm to have been fulfilled. Even in the subgroup of people with viral load greater than 1000 copies per mL and presence of drug mutations to tenofovir and lamivudine at baseline, switching to tenofovir, lamivudine, and dolutegravir was predicted to bring benefits compared with the policies of tenofovir, lamivudine, and dolutegravir dependent on viral suppression, as members of this subgroup must await fulfilment of the viral load failure criteria and subsequent switch to zidovudine, lamivudine, and dolutegravir. These benefits occur despite the fact that with the tenofovir, lamivudine, and dolutegravir for all policy, the proportion of people on ART with viral suppression over 1 year, 5 years, and 20 years is predicted to be low (55% on average over 5 years) because of the higher tendency for poor adherence in this subgroup and the potential for dolutegravir resistance to emerge. Overall, dolutegravir resistance (transmitted or acquired) is predicted to be present in 6·7% of people over a 20 year period under the policy of tenofovir, lamivudine, and dolutegravir for all compared with 4·4% under the policy of tenofovir, lamivudine, and dolutegravir dependent on viral suppression only. Our findings were consistent in several sensitivity analyses, including one in which we assumed that lamivudine and tenofovir had no effect on viral replication in the presence of the Met184Val and Lys65Arg mutations, and one over a 50 year time horizon.

More children are predicted to be prevented from acquiring HIV through mother-to-child transmission than are born with neural tube defects with a tenofovir, lamivudine, and dolutegravir for all policy. When calculating DALYs in children born with HIV, we assumed availability of good HIV diagnosis and treatment services in infants meaning less poor consequences for an HIV infection in a child (0·1 DALYs per year) than for a child born with a neural tube defect (1 DALY per year). Without such good availability of diagnosis and treatment services, the DALY consequences of a child born with HIV would be worse and the benefits of a policy of tenofovir, lamivudine, and dolutegravir for all greater.

Our study has limitations. As for any cost-effectiveness analysis over a long time horizon, we relied on a model for predictions of the long-term effect of the alternative policies. We made assumptions about the benefits and harms associated with different ART regimens on the basis of available data, which included different levels of uncertainty. The reports^9^ of neural tube defects in infants born to women using dolutegravir are the first indication of this risk and planned further follow-up of women who have been similarly exposed could reduce uncertainty over this risk. This situation emphasises that studies of novel drugs during the development phase and beyond—including in women before conception, during pregnancy, and postpartum—are crucial to support future decision making. Evidence of the antiretroviral effects of dolutegravir is more extensive, including recent data from a randomised trial in Cameroon.^32^ Our results are robust to modifications in assumptions within plausible levels of uncertainty, which supports our main conclusions. We recognise that our assumption that women can access contraception from the time of intention to have no (more) children does not reflect reality in many settings, and partly accounted for this by considering both an 80% and 50% effectiveness of contraception. We did not consider a policy option in which women could move between efavirenz and dolutegravir between pregnancies because we considered that this would be unrealistic to implement.

Our results could be used to inform relevant constituencies about the risks and benefits of alternative dolutegravir-containing ART regimen policy options, and to help gain an informed representative view from people with HIV that can be considered during development of ministry of health policies. If ministries of health decide to give dolutegravir to women who might become pregnant without adequate consultation and it then becomes clear that there is a real risk of neural tube defects, this could undermine confidence in treatment and in the ministry of health, although the extent to which ART uptake could be affected is difficult to quantify. However, we recognise that there will be substantial challenges in achieving such effective consultation.

In conclusion, the benefits of transition to tenofovir, lamivudine, and dolutegravir for all substantially outweighed any risks when using a standard DALY framework to compare health outcomes from a public health perspective.

## Data sharing

Model programs and simulations are accessible in Figshare.
